# Circadian rhythm and gestational diabetes: working conditions, sleeping habits and lifestyle influence insulin dependency during pregnancy

**DOI:** 10.1007/s00592-021-01708-8

**Published:** 2021-04-10

**Authors:** Friederike Weschenfelder, Karolin Lohse, Thomas Lehmann, Ekkehard Schleußner, Tanja Groten

**Affiliations:** 1 Department of Obstetrics, University Hospital Jena, Am Klinikum 1, 07747 Jena, Germany; 2 Unit Neonatology, Department of Paediatrics, University Hospital Jena, Jena, Germany; 3 Institute of Medical Statistics and Computer Science, University Hospital Jena, Friedrich Schiller University Jena, Jena, Germany

**Keywords:** Circadian rhythm, Gestational diabetes, Sleep, Insulin treatment, Work conditions

## Abstract

**Objective:**

Management of gestational diabetes (GDM) is currently changing toward a more personalized approach. There  is a growing number of GDM patients requiring only a single dose of basal insulin at night to achieve glucose control. Well-known risk factors like obesity, parity and family history have been associated with GDM treatment requirements. Sleep quality and lifestyle factors interfering with the circadian rhythm are known to affect glucose metabolism. The aim of this study was to investigate the impact of such lifestyle factors on insulin requirement in GDM patients, in particular on long-acting insulin to control fasting glucose levels.

**Research design and methods:**

A total of 805 patients treated for GDM between 2012 and 2016 received a study questionnaire on lifestyle conditions. Sleep quality and work condition categories were used for subgroup analysis. Independent effects on treatment approaches were evaluated using multivariate regression.

**Results:**

In total, 235 (29.2%) questionnaires returned. Women reporting poor sleep conditions had higher pre-pregnancy weight and BMI, heavier newborns, more large for gestational age newborns and higher rates of hyperbilirubinemia. Treatment requirements were related to sleep and work condition categories. Multivariate regression for ‘Basal’ insulin-only treatment revealed an adjOR 3.4 (CI 1.23–9.40, *p * <  0.05) for unfavorable work conditions and adjOR 4.3 (CI 1.28–14.50, *p * <  0.05) for living with children.

**Conclusions:**

Our findings suggest that external stressors like unfavorable work conditions and living with children are independently associated with the necessity of long-acting insulin at night in GDM patients. Thus, fasting glucose levels of pregnant women presenting with such lifestyle conditions may be subject to close monitoring.

**Supplementary Information:**

The online version contains supplementary material available at 10.1007/s00592-021-01708-8.

## Introduction

Treatment of gestational diabetes (GDM) is currently changing from uniform standard therapy regimes toward a more individualized and patient-orientated management. Leaving rigid therapy standards, we observed that, where insulin treatment is inevitable, a number of women were sufficiently treated by injecting a single dose of long-acting basal insulin at night. Accordingly, the recently updated German GDM guidelines included adaptations reflecting the awareness of this special subgroup by recommending to start insulin when 50% of the fasting glucose measurements are exceeding the target range of 5.0–5.3 mmol/l (31–34 mmol/mol) [1].

Predictors for insulin therapy during pregnancy in previous studies included values of the 75 g oral glucose tolerance test (oGTT) and maternal anthropometrics showing that mostly elevated prepregnancy maternal BMI and levels of fasting glucose, 2-h blood glucose and HbA1c at time of diagnosis were associated with the requirement of insulin treatment during pregnancy [2–4]. Recently, besides the well-known risk factors obesity, parity and family history of diabetes, effects of lifestyle have also been put into focus to impact on the development of diabetes mellitus [5–7]. During the last decade, sleep habits and other lifestyle factors interfering with the circadian rhythm have been shown to increase glucose levels and the risk of diabetes in general [8–10]. There is substantial empirical evidence from studies conducted decades ago indicating that short sleep alters glucose metabolism [11]. Stamatakis et al. provide potential mechanisms through which sleep fragmentation across any sleep stages alters glucose metabolism by showing that interrupted sleep leads to higher morning cortisol levels [12] accompanied by an increase in blood sugar levels. Thus, it is reasonable to assume that such risk factors for diabetes also have a potential impact on the need for insulin treatment in GDM.

The aim of our study was to investigate the impact of lifestyle conditions like shift work, sleep quality, eating habits and occupational satisfaction on GDM treatment necessities. We hypothesized that the above-mentioned conditions associated with maternal chronodisruption may in particular affect the requirement of basal insulin treatment at night.

## Research design and methods

### Study population

The primary cohort of this study consist of 805 GDM patients of our outpatient department for diabetes and pregnancy. Singleton GDM pregnancies, consulted from January 1, 2012, to December 31, 2016, were included in our study. The diagnosis of GDM in this cohort was based on the IADPSG and WHO-2013 criteria [13, 14]. Diabetes care was provided according to the German S3 guidelines published in 2011 [15] and provided by our hospital-based outpatient department. Patients performed self-monitoring of blood glucose (SMBG) four times or in case of insulin treatment seven times per day. The cohort was monitored every four weeks in case of diet control and fortnightly if insulin treatment was needed. Ethical approval was given by the local Ethical Committee of the Friedrich Schiller University, Jena, Germany (5280-09/17).

### Study questionnaire

To determine the effect of the circadian rhythm, a questionnaire was developed (see Supplement S1). All 805 women were contacted and received the questionnaire by mail at the latest 5 years after the GDM index pregnancy. The questionnaire included questions about work and life conditions before and during the index pregnancy. Using dichotomous variables (Yes/No), women were asked for employment during and before the index pregnancy, working fulltime, working overtime, shift works, night shifts, irregular meals during work and occupational satisfaction. Some variables were additionally analyzed categorical: regular meals (never/rare/mostly/always) and occupational satisfaction by asking if they would choose the same profession again (yes/probably/probably not/no). We defined the combined variable ‘unfavorable work conditions’ as a dichotomous variable for cases where either shift work, night shifts or more than 2–5 h overtime working per week before pregnancy was recorded. This variable was then used for subgroup analysis.

To assess the individual sleeping conditions, women were asked about insomnia at the time of pregnancy, sleep disturbances on a regular basis (e.g., by street noise, neighbors, children), hours of sleep per night before pregnancy (analyzed categorically < 5 h, 5 h to < 7 h, > 7 h). Cases where either sleeping disruptions, insomnia or an average sleeping time below 5 h had been documented were defined as a combined variable ‘poor sleep quality’ that was also used for subgroup analysis.

Questions about social and patient’s history included marital status (single, married, divorced, permanent relationship) and the dichotomous variables: kids living within the same household.

### Study data collection

Basic characteristics and patient’s history and family history were retrieved from patient records. BMI was calculated from maternal height and the documented prepregnancy weight and grouped according to the definitions of the World Health Organization [16]. Gestational weight gain (GWG) was calculated as the difference of the prepregnancy weight and the last documented weight during pregnancy. Maternal blood pressure and HbA1c levels according to IFCC or NGSP/DCCT standard were determined on a four weekly basis in a standardized setting. Change of HbA1c was calculated by using the first HbA1c value at diagnosis and the last HbA1c obtained before delivery. Treatment groups during pregnancy were ‘Diet,’ ‘Bolus,’ ‘multiple daily injections (MDI)’ and ‘Basal’, according to their individual treatment requirements of dietary therapy only, short-acting insulin injected with meals, multiple daily injections of long- and short-acting insulin, and injection of long-acting (basal-) insulin only.

Fetal biometry was performed using standardized anatomic views according to ISUOG guidelines [17]. AC percentiles were calculated using Snijders [18] and EFW percentiles using Hadlock’s formula for EFW [19].

Perinatal outcome data included fetal birth weight, birth weight percentiles according to Voigt et al. [20]. Neonates were also grouped into LGA (large for gestational age; fetal growth above 90th percentile) and SGA (small for gestational age; fetal growth below the 10th percentile) according to gestational age and sex. Further neonatal outcome data were 5-min Apgar score, postnatal admission to NICU, neonatal hypoglycemia and hyperbilirubinemia. Outcome data were retrieved from the standardized nationwide used perinatal documentation systems of our University hospital and patient’s maternity records.

### Statistical analysis

Statistical analysis was performed with SPSS 24.0 (IBM Corp. Released 2016. IBM SPSS Statistics for Windows, Version 24.0. Armonk, NY: IBM Corp). We used Chi-square test or Fisher exact test to compare categorical data. Most of the continuous data were not normally distributed; therefore, we used the median and interquartile range for data presentation and description. Nonparametric tests were performed to compare continuous data between subgroups: ‘Good sleep quality’ versus ‘Poor sleep quality’ and ‘Good work conditions’ vs. ‘Unfavorable work conditions,’ respectively. Adjusted odds ratios (ORs) for estimating the association between treatment methods and BMI, parity, history of GDM, ‘unfavorable work conditions’, irregular meals, occupational satisfaction, ‘poor sleep quality’, living with children were determined using logistic regression. ORs with 95% confidence interval (CI) are presented. Only significant variables are presented in the tables. The other non-significant covariates of the statistical analysis are presented in footnotes. Regression models were tested for an overall predictive value using Omnibus-Tests. Only significant overall predictive models are presented [21]. A *p*-value < 0.05 was considered to indicate statistical significance (2-tailed).

## Results

In total, 805 women were contacted and received the study questionnaire; 235 (29.2%) women returned their questionnaires and consented in study participation. The number of returned questionnaires was uniformly distributed over the years of the survey period ranging from 14.5 to 27.2%.

### Baseline characteristics

Table 1 shows the baseline characteristics at time of GDM diagnosis (median 26 weeks of gestation (IQR 23; 28). Sleep subgroup analysis did not reveal differences concerning maternal age at diagnosis, pre-pregnancy BMI categories, systolic and diastolic blood pressure, family history of diabetes, thyroid disorders, fetal ultrasound parameters at diagnosis and 75 g oGTT values. There were significant differences of pre-pregnancy weight and BMI depending on sleep quality. Women with poor sleep quality were significantly heavier than women with ‘good sleep quality’ (77 kg (IQR 68–91.5) vs. 71 kg (63–86), *p* < 0.05) and showed higher BMI values before pregnancy (27.7 kg/m2 (23.7–33.9) vs. 26.1 kg/m2 (23.1–30.4)). Women with ‘‘good sleep quality’’ were also more likely to be Nulliparous (Median 0; IQR 0–1) compared to the ‘bad sleep quality’ group (Median 1; IQR 0–1) (*p* < 0.05).

**Table 1:** Baseline characteristics of the total cohort and the sleep and work condition subgroups

Variable	Total cohort (*n* = 235)	‘Good sleep quality’ (*n* = 144; 61.3%)	‘Poor sleep quality’† (*n* = 91; 38.7%)	*p*	‘Good work conditions’ (*n* = 115; 50.2%)	‘Unfavorable work conditions’‡ (*n* = 114; 49.8%)	*p*
Age in years	32 (28–35)	31 (28–35)	32 29 35	n.s.	33 (29–36)	31 (28–33)	** < 0.05**
Parity	1 (0–1)	0 (0–1)	1 (0–1)	** < 0.05**	1 (0–1)	0 (0–1)	** < 0.01**
Prepregnancy weight in kg	73.0 (63.8–88.6)	71 (63–86)	77 (68–91.5)	** < 0.05**	74 (63.8–87.2)	72.5 (63–89.5)	n.s.
Prepregnancy BMI in kg/m^2^	26.6 (23.1–31.3)	26.1 (23.1–30.4)	27.7 (23.7–33.9)	** < 0.05**	26.6 (23.0–31.1)	26.3 (23.1–31.5)	n.s.
Prepregnancy BMI categories				n.s.			n.s.
< 18.5 kg/m^2^)(underweight)	0.9%	1.4%	–		0.9%	0.9%	
18.5–24.9 kg/m^2^ (normal weight)	39.3%	43.8%	31.9%		39.5%	40.4%	
25–29.9 kg/m^2^ (overweight)	28.2%	27.1%	29.7%		28.9%	27.2%	
≥ 30 kg/m^2^ (obesity)	31.6%	27.8%	37.4%		30.7%	31.6%	
Systolic BP at diagnosis	122 (115–130)	120 (144–130)	125 (115–132)	n.s.	124 (115–130)	121 (114–130)	n.s.
Diastolic BP at diagnosis	80 (70–85.3)	80 (70–85)	79 (70–86)	n.s.	80 (70–85)	80 (70.5–87.5)	n.s.
History of GDM	10.3%	7.6%	14.3%	n.s.	9.6%	9.7%	n.s.
Thyroid disorders	23.6%	21.5%	26.4%	n.s.	23.9%	22.8%	n.s.
Cardiovascular disorders	12.4%	10.4%	15.4%	n.s.	10.6%	13.2%	
Marital status				n.s.			n.s.
Single	1.7%	1.4%	2.2%		1.8%	1.8%	
Married	43.3%	38.7%	50.5%		47.4%	39.8%	
Divorced	1.3%	1.4%	1.1%		0.9%	1.8%	
Permanent relationship	53.6%	58.8%	46.2%		50%	56.6%	
Family history of diabetes	54.1%	54.9%	51.6%	n.s.	49.1%	59.3%	n.s.
Percentile of EFW at diagnosis	42 (24–72)	41 (24–73)	50 (27–68)	n.s.	42 (27–66)	44 (23–74)	n.s.
Percentile of AC at diagnosis	59 (40.5–79)	55 (40–77)	61.5 (43.3–80)	n.s.	56 (43–77)	62 (40–80)	n.s.
HbA1c at diagnosis in %	5.3 (5–5.4)	5.3 (5–5.4)	5.3 (5–5.5)	n.s.	5.3 (5–5.4)	5.3 (5–5.5)	n.s.
HbA1c at diagnosis in mmol/mol	34 (31–36)	34 (31–36)	34 (31–39)	n.s.	34 (31–36)	34 (31–39)	n.s.
75 g ogTT (in mmol/l)							
Fasting	5.2 (4.7–5.6)	5.1 (4.7–5.6)	5.3 (4.9–5.6)	n.s.	5.2 (4.7–5.7)	5.1 (4.7–5.5)	n.s.
1 h	9.7 (8.3–10.8)	9.7 (8.6–10.8)	9.7 (7.6–10.9)	n.s.	9.6 (8.3–11)	9.7 (8.2–10.5)	n.s.
2 h	7.5 (6.4–8.6)	7.5 (6.6–8.6)	7.2 (5.9–8.5)	n.s.	7.5 (6.6–8.7)	7.3 (6.3–8.6)	n.s.
Diagnosis based on isolated fasting	37.4%	35.6%	40.2%	n.s.	38%	38%	n.s.
Elevated fasting	65.4%	52.8%	63.7%	n.s.	54%	65.7%	n.s.
Elevated 1 h value	44.8%	45.2%	44.3%	n.s.	45.5%	42.9%	n.s.
Elevated 2 h value	28.7%	28.2%	29.5%	n.s.	26.5%	30.6%	n.s.
Employment during pregnancy	94.9%	96.5%	92.3%	n.s.	96.5%	98.2%	n.s.
Working fulltime	70.2%	73.8%	64.3%	n.s.	67.9%	72.6%	n.s.
Would not choose job again	21.5%	14.8%	32.6%	** < 0.01**	85.5%	71.9%	** < 0.05**
Working overtime	46.7%	40%	56.7%	** < 0.05**	14.1%	75.3%	** < 0.01**
Irregular meals	27%	21%	36.8%	** < 0.05**	13%	41.2%	** < 0.01**
Shift work	31%	71.3%	65.1%	n.s.	–	62.3%	** < 0.01**
Night shifts	19.2%	16.8%	23.3%	n.s.	–	38.6%	** < 0.01**
Insomnia	23.2%	0	60%	** < 0.01**	15.8%	28.9%	** < 0.05**
Average sleeping hours							
< 5 h	2.6%	0%	6.7%	** < 0.01**	2.6%	0.9%	** < 0.05**
5–7 h	54.7%	47.9%	65.6%		47.4%	63.2%	
> 7 h	42.7%	52.1%	27.8%		50%	36.0%	
Sleep interruptions	38.7%	0%	72.5%	** < 0.01**	29.8%	25.7%	n.s.
Living with kids	67.2%	60.3%	78.1%	** < 0.05**	78.5%	54.4%	** < 0.01**

Data are n (%) or median (interquartile range) unless otherwise specified

*AC* abdominal circumference, *BMI* body mass index, *BP* blood pressure, *EFW* estimated fetal weight, *GDM* gestational diabetes, *MDI* multiple daily injections

p* Comparison of good versus ‘poor sleep quality’ subgroups; *p*† comparison of good versus ‘unfavorable work conditions’; *p * <  0.05 is significant and bold

^‡^Variable includes cases with either shift work, night shifts or more than 2–5 h overtime working per week. †Variable includes cases with either sleeping disruptions, insomnia or an average sleeping time below 5 h

Baseline characteristics in the work subgroup analysis revealed that women with ‘unfavorable work conditions’ were significantly younger (Median 31; IQR 28–33) and were mostly nulliparous (Median 0; IQR 0–1) compared to the ‘good work conditions’ subgroup (Median age 33, IQR 29–36) and Parity (1; IQR 0–1). Besides that, none of the other variables differed between subgroups.

Women with ‘poor sleep quality’ were more likely to work overtime (56.7% vs. 40%; *p* < 0.05), had higher rates of dissatisfaction with their occupation (32.6% vs. 14.8; *p* < 0.01), had less regular meals (36.8% vs. 21%; *p* < 0.05) and mostly lived with children in the same household (78.1% vs. 60.3%, *p* < 0.05) (see Table 1).

Women with ‘unfavorable work conditions’ showed higher rates of insomnia (28.9% vs. 15.8%; p < 0.05), lower rates of average sleeping hours above 7 h (36% vs. 50%, *p* < 0.05). A significantly lower rate of women living with children was found in the ‘unfavorable work conditions’ subgroup (54.4% vs. 78.5%; *p* < 0.01).

### Pregnancy and perinatal outcome

Pregnancy characteristics and perinatal outcome are summarized in Table 2. No difference in HbA1c at delivery, gestational weight gain, pregnancy complications, pregnancy-related hypertensive disorders, necessity of induction of labor (IOL), gestational age at delivery or mode of delivery was seen in both subgroup analyses. We did see differences in the treatment groups depending on sleep quality. Women with poor sleep quality were more likely to be treated with basal insulin, either solely or in combination with short-acting insulin (MDI) (overall 42.9% vs. 27.1%; *p* < 0.05). Children born by mothers with ‘poor sleep quality’ were significantly heavier (3605 g vs. 3395 g; 0.01) more frequent LGA (20.3% vs. 7.5%; *p* < 0.05) and showed higher rates of hyperbilirubinemia (43.1% vs. 18.5%; *p* < 0.01).

**Table 2:** Pregnancy characteristics and perinatal outcome of the total cohort and the sleep and work condition subgroups

Variable	Total cohort (*n* = 235)	‘Good sleep quality’ (*n* = 144; 61.3%)	‘Poor sleep quality’† (*n* = 91; 38.7%)	p*	‘Good work conditions’ *N* = 115; 50.2%	‘Unfavorable work conditions’‡ *n* = 114; 49.8%	*p*†
Hba1c at delivery in %	5.5 (5.2–5.7)	5.5 (5.3–5.7)	5.5 (5.2–5.7)	n.s.	5.4 (5.2–5.7)	5.5 (5.3–5.7)	n.s.
Hba1c at delivery in mmol/mol	37 (33–39)	37 (34–39)	37 (33–39)	n.s.	36 (33–39)	37 (34–39)	n.s.
Hba1c changes in %	0.2 (0–0.4)	0.2 (0–0.4)	0.2 (0–0.4)	n.s.	0.2 (0–0.4)	0.1 (0–0.4)	n.s.
HbA1c changes in mmol/mol	2 (0–4)	2 (0–4)	2 (0–4)	n.s.	2 (0–4)	1 (0–4)	n.s.
Need for insulin	43.8%	41%	48.4%	n.s.	35.7%	50%	** < 0.05**
Treatment methods							
Diet	56.2%	59%	51.6%	** < 0.05**	64.3%	50%	n.s.
Bolus	10.6%	13.9%	5.5%		10.4%	10.5%	
Basal	16.6%	13.2%	22%		11.3%	21.1%	
MDI	16.6%	13.9%	20.9%		13.9%	18.4%	
Insulin IU/kg	0.31 (0.17–0.48)	0.33 (0.19–0.51)	0.26 (0.16–0.38)	n.s.	0.34 (0.2–0.5)	0.27 (0.17–0.47)	n.s.
GWG in kg	12 (8–16.6)	12 (8.1–16.5)	12.7 (8–16.6)	n.s.	11.9 (8–6.1)	13 (8.1–16.8)	n.s.
Pregnancy complications	31.3%	30.5%	32.5%	n.s.	32.6%	30.9%	n.s.
Pre-eclampsia/ PIH/ HELLP	8.3%	6.2%	11.3%	n.s.	6.4%	10.5%	n.s.
IOL	39%	37.6%	41%	n.s.	36.7%	40.6%	n.s.
C-section	32%	34.7%	28%	n.s.	27.1%	36.8%	n.s.
Birth weight	3500 (3135–3825)	3395 (3040–3710)	3605 (3310–3900)	** < 0.01**	3540 (3220–3840)	3462 (3062–3803)	n.s.
GA at delivery	39 (38–40)	39 (38–40)	39 (38–40)	n.s.			
Voigt’s percentile	58 (31–78)	53 (28–73)	62 (35–86)	** < 0.01**	65 (37–80)	51 (28–73)	n.s.
SGA	6%	7.5%	3.8%	n.s.	5.3%	6.9%	n.s.
LGA	12.6%	7.5%	20.3%	** < 0.05**	13.8%	10.9%	n.s.
5 min APGAR	9 (9–10)	9 (9–10)	9 (9–10)	n.s.	9 (9–10)	9 (9–10)	n.s.
pH	7.26 (7.20–7.31)	7.26 (7.21–7.33)	7.26 (7.19–7.3)	n.s.	7.25 (7.19–7.31)	7.26 (7.21–7.31)	n.s.
NICU admission	7%	7.5%	6.3%	n.s.	6.9%	7.4%	n.s.
hyperbilirubinemia	27.3%	18.5%	43.1%	** < 0.01**	21.5%	31.6%	n.s.
hypoglycemia	1.7%	3%	–	n.s.	1.2%	2.3%	n.s.

Data are *n* (%) or median (interquartile range) unless otherwise specified

*GA* gestational age, *GWG* gestational weight gain, *IOL* induction of labor, *LGA* large for gestational age, *NICU* neonatal intensive care unit, *PIH* pregnancy induced hypertension, *SGA* small for gestational age

p* Comparison of good versus ‘poor sleep quality’ subgroups; *p*† comparison of good versus. ‘unfavorable work conditions’; *p* < 0.05 is significant and bold

The work condition subgroup analysis revealed women with ‘unfavorable work conditions’ to be more likely develop a need for insulin (50% vs. 35.7%; *p* < 0.05).

### Working conditions, sleep quality and stress

There were no significant differences in employment status, fulltime working, irregular meals, job satisfaction, overtime working, shift work and nightshifts within the four therapy groups (Table 3). Nevertheless, we could see a trend toward most overtime working (69.2%), most nightshift working (27%) and lowest job satisfaction (31.6%) in the ‘Basal’ subgroup. Consequently, the highest frequency of the combined parameter ‘unfavorable work conditions’ was found in this subgroup with 64.9%.

**Table 3:** Working conditions and sleep quality depending on GDM treatment

Variable	Total cohort (*n* = 235) (%)	Diet (*n* = 132) (%)	Bolus (*n* = 25) (%)	MDI (*n* = 39) (%)	Basal (*n* = 39) (%)	p*
Employment during pregnancy	94.5	95.5	96.0	92.3	94.9	n.s.
Working fulltime	70.5	67.2	66.7	75.0	78.4	n.s.
Would not choose job again	16.6	19.4	8.3	27	31.6	n.s.
Working overtime	46.7	39.5	45.5	48.5	69.2	n.s.
Irregular meals	27	29.8	25	16.2	28.9	n.s.
Shift work	31	29	25	40.5	32.4	n.s.
Night shifts	19.2	17.6	8.3	24.3	27	n.s.
‘Unfavorable work conditions’†	49.7	43.5	50	56.8	64.9	n.s.
Insomnia	23.2	21.4	20	33.3	21.1%	n.s.
Average sleeping hours						n.s.
< 5 h	2.6	2.3	0	2.6	5.1	
5–7 h	54.7	48.9	68	56.4	64.1	
> 7 h	42.7	48.9	32	41.0	30.8	
Sleep interruptions	28.3	24.6	12	41	38.5	< 0.05
‘Poor sleep quality’‡	38.7%	35.6	20	48.7	51.3	< 0.05
Living with kids	67.2%	62.5	72.7	79.3	67.6	n.s.

Data are n (%)

*p—Comparison of the four subgroups using Wilcoxon test, *p *  <   0.05 is significant and bold

†Variable includes cases with either shift work, night shifts or more than 2–5 h overtime working per week. ‡Variable includes cases with either sleeping disruptions, insomnia or an average sleeping time below 5 h

We did not see a difference in preexisting insomnia or average sleeping hours. A significant group difference could be seen for frequency of sleep interruptions that was more common in the ‘MDI’ (41%) and ‘Basal’ subgroup (38.5%) compared to 24.6% in the ‘Diet’ subgroup or 12% in the ‘Bolus’ subgroup (*p* < 0.05). Accordingly, poor sleep quality was significantly more frequent in these two groups with 51.3% in the ‘Basal’ and 48.7% in the ‘MDI’ compared to 35.6% in the ‘Diet’ and 20% in the ‘Bolus’ subgroup (*p* < 0.05). There were no differences in the rates of women living with kids.

### Association of circadian measures with requirements of GDM treatment

We included the following variables in a multivariate regression model: BMI, parity, history of GDM, ‘unfavorable work conditions’, irregular meals, job satisfaction, ‘poor sleep quality’ and living with children as presented in Table 4. First, regression model tested the impact of the above-mentioned variables on overall necessity of insulin treatment during pregnancy. BMI (adjOR 1.08; CI 1.02–1.15), parity (adjOR 1.88; CI 1.13–3.11) and ‘unfavorable work conditions’ (adjOR 3.40; CI 1.64–7.06) revealed to be of significant impact.

**Table 4:** Multivariate analysis

Variable	Independent variables with significant influence	OR	CI	CI	*p*
Necessity of any insulin	BMI	1.08*	1.02	1.15	0.006
	Para	1.88*	1.13	3.11	0.014
	‘Unfavorable work conditions’	3.40*	1.64	7.06	0.001
Necessity of bolus insulin	Para	2.21*	1.32	3.71	0.003
Necessity of basal insulin	BMI	1.09*	1.03	1.16	0.003
	‘Unfavorable work conditions’	2.85*	1.33	6.11	0.007
Necessity of exclusively basal insulin	‘Unfavorable work conditions’	3.40*	1.23	9.40	0.018
	Living with children	4.31*	1.28	14.50	0.018

Variables included: BMI, parity, History of GDM, ‘unfavorable work conditions’, Irregular Meals, Dissatisfaction with occupation, ‘poor sleep quality’, living with children

Prior models tested significant via Omnibustest (*p * <  0.05)

The necessity of bolus insulin as a treatment option was only significantly associated with parity (adjOR 2.21; CI 1.32–3.71). Basal insulin as a part of the GDM treatment was independently associated with pre-pregnancy BMI (adjOR 1.09; CI 1.03–1.16) and ‘unfavorable work conditions’ (adjOR 2.85; CI 1.33–6.11). The necessity for exclusively basal insulin treatment was significantly impacted by ‘unfavorable work conditions’ (adjOR 3.40; CI 1.23–9.40) and by children living within the same household (adjOR 4.31; CI 1.28–14.50).

## Discussion

Main aim of this study was to analyze the potential effects of lifestyle on insulin dependency in GDM pregnancies. Our findings suggest that ‘unfavorable work conditions’ and ‘poor sleep quality’ as a stressor on maternal chronodisruption affect insulin dependency and treatment necessities in GDM pregnancies.

Most of the published studies on GDM-related topics so far do not differentiate treatment approaches regarding insulin administration within the group of insulin treated patients. Individualized patient-oriented treatment approaches carried out at our department resulted in four heterogeneous treatment groups. The ‘Diet’ group was sufficiently controlled by dietary changes and physical activity, the ‘Bolus’ group only required short acting insulin to control postprandial glucose levels, the ‘MDI’ group required classical insulin treatment including long and short acting insulin and individuals in the ‘Basal’ group showed constantly elevated fasting glucose levels requiring a single dose of long-acting insulin at night. All groups revealed equal sufficient glucose control and perinatal outcome. (see Supplemental Table S2).

‘Poor sleep quality’ (including sleeping disruptions, insomnia or an average of sleeping hours below 5) in our GDM cohort was significantly associated with higher maternal pre-pregnancy weight and BMI (27.7 kg/m^2^ vs. 26.1 kg/m^2^ in the “good-sleep” group). This result matches the findings of Mokhlesi et al. that people reporting short sleep and shift work had higher BMI [8].

Coincidentally with the worldwide increase in non-communicable diseases in the last few decades, a rising incidence of GDM cases up to 11% and higher using IADPSG criteria has been reported [22]. As recently shown by Lateef et al., lifestyle conditions affect young women during their fertile period of life including sleep deprivation and circadian misalignments leading to altered gonadotropin and sex steroid secretion interfering with female fertility [23]. McMullan et al. also found evidence that nocturnal melatonin secretion was independently and inversely associated with insulin resistance in young non-diabetic women [24] hypothetically leading to higher numbers of GDM pregnancies or even influencing the severity of a GDM disease.

We could show that the ‘poor sleep quality’ was significantly more frequent in the ‘Basal’ subgroup with (51.3%; *p* < 0.05). ‘MDI’ and ‘Basal’ subgroup showed the highest frequency of sleep interruptions (41% and 38.5%; *p *< 0.05). These results match the recently published study of Mokhlesi et al. They were able to show that self-reported short but also too long sleep were both associated with adverse measures of glycemia in a cohort of 962 overweight or obese adults with prediabetes or recently diagnosed, untreated type 2 diabetes [8].

According to the hypothesis that ‘poor sleep quality’ leads to elevated nocturnal glucose, we assume this might be the reason for the significantly higher fetal birth weight (3605 g vs. 3395 g), higher number of LGA newborns (20.3% vs. 7.5%) and also the higher number of hyperbilirubinemia (43.1% vs. 18.5%) in the ‘‘poor sleep quality’’ group. Complications are well known to be caused by poor maternal blood glucose control. Law et al. published a CGM-based glucose analysis of GDM pregnancies and showed that mothers of LGA infants ran significantly higher glucose overnight. Consequently, the authors recommend better detection and controlling of nocturnal glucose levels to prevent fetal overnutrition [25]. It has also been shown that even first trimester glucose is significantly and independently associated with LGA newborns [26]. Thus, elevated levels of fasting glucose should gain more attention in the management of GDM. However, the higher fetal birth weight in the ‘poor sleep quality’ group could also be explained by maternal prepregnancy BMI which is well known to also have an impact on fetal growth in GDM and non-GDM pregnancies [27–29].

Obese women are known to suffer more often sleep apnea and other sleep disorders [30, 31]. Therefore, it is a chicken or the egg dilemma, to decide if either poor sleep causes higher BMI leading to LGA or if the higher BMI causes ‘poor sleep quality’ leading to fetal overgrowth.

Subgroup analysis on work conditions revealed that women with ‘unfavorable work conditions’ showed a significantly higher need for insulin treatment during pregnancy (50% vs. 45.7%). We could also see a trend to higher rate of women needing basal insulin, either solely or combined (MDI) (39.5% vs. 25.2%), in the group with ‘unfavorable work conditions’; nevertheless, this finding lacked statistical significance. In this subgroup, we could also find higher rates of insomnia (28.9% vs. 15.8%). These findings match the result of Ahmed et al. They found an association between poor sleep quality during pregnancy, the work schedule and women’s moods. They recommend interventions targeting mental health and lifestyles to improve sleep quality during pregnancy to improve maternal and fetal well-being [32].

Multivariate analysis showed that the need for insulin treatment during pregnancy was significantly independently affected by prepregnancy BMI (adjOR 1.08), parity (adjOR 1.88) and ‘unfavorable work conditions’ (adjOR 3.40). BMI and parity are already known to affect insulin treatment necessity in GDM pregnancies. As far as we know the effect of poor work conditions has not been shown yet. Since we could clearly show that ‘unfavorable work conditions’ are related to ‘poor sleep quality,’ we speculate that the higher cortisol levels as a physiological response to stress might well be a reason for the insulin resistance leading to the need for insulin treatment. Looking at the women treated with basal insulin (either solely or as a combination in MDI) BMI (adj. OR 1.09) and work conditions (adj. OR 2.85) stayed relevant and significant influence factors. Surprisingly, the need for exclusively basal insulin treatment was not affected by maternal BMI but by ‘unfavorable work conditions’ (adjOR3.40) and living with kids (adjOR 4.31). Our hypotheses is that, since kids, especially younger kids, tend to cause sleep interruptions, the need for insulin at night in this group might increase due to higher cortisol levels as well.

Since ‘unfavorable work conditions’ and ‘poor sleep quality’ seem to be highly correlated, this could explain why sleep quality does not appear as an individual risk factor in the multivariate analysis. Nevertheless, our data strongly implicate that sleep quality has a robust impact on the necessity of insulin treatment, in particular on the need of basal insulin.

### Strength and limitations

A strength of our study is the comparison of a large number of GDM cases treated at only one outpatient clinic following to the same treatment standards. Nevertheless, there are some limitations of the present study that might include the retrospective survey design and the consequential time difference between GDM pregnancy and the survey. The questionnaire used for this study was for preliminary evaluation only; therefore, we did include a higher number of potential influence factors, well knowing that this leads to a loss of specificity for certain variables. Concerning the sample size calculation, it is recommended that the sample should include at least ten events per independent variable in order to get unbiased estimates in a multivariate binary logistic regression model [33]. In our primary analyses, 103 events of insulin treatment are observed, which is sufficient for the analyses of eight independent variables according to the rule mentioned above. Nevertheless, this does not apply for different treatment subgroups since these events are rarer. The reliability of the electronic records concerning the patient data is also a limitation. To strengthen our hypothesis that sleep interruptions and everyday stress lead to high cortisol levels interfering with blood glucose, measurement of cortisol levels in our patients would have been of particular interest. Due to the retrospective character of this analysis our study lacks this information.

## Conclusion

Our study provides evidence that external stressors like ‘unfavorable work conditions’ (including shift work, night shifts and working overtime) and living with children also affect the need for insulin treatment during a GDM pregnancy. Since half of the women with exclusively basal insulin treatment were diagnosed with GDM by isolated elevated fasting glucose only, they might have been missed to be diagnosed by the 50 g glucose challenge test (GCT) screening. Therefore, our findings tentatively suggest that women with the here described lifestyle-related risk factors benefit from primarily being tested with a 75 g oGTT or, at least, would benefit from additional determination of fasting glucose levels. External stressors should get more attention as potential risk factors for development of GDM and insulin dependency.

## Supplementary Information

Below is the link to the electronic supplementary material.

Supplementary file1 (PDF 189 KB)

Supplementary file2 (DOCX 26 KB)

## Data Availability

The datasets used and/or analyzed during the current study are available from the corresponding author on reasonable request.
