# Supplementary material for: Circadian rhythm and gestational diabetes: working conditions, sleeping habits and lifestyle influence insulin dependency during pregnancy
Source: Acta Diabetol. 2021 Apr 10;58(9):1177–86. doi: 10.1007/s00592-021-01708-8 (PMC8316165; doi:10.1007/s00592-021-01708-8)
Supplement: Supplementary file 1 — Supplementary file1 (PDF 189 KB) [file 592_2021_1708_MOESM1_ESM.pdf]

## Study Questionnaire

**Questionnaire Number:**

Circadian rhythm and gestational diabetes
Lifestyle, working conditions and sleeping habits influence insulin dependency during pregnancy

**Where you employed before the pregnancy?**

- ☐ Yes
- ☐ No

If yes, what was your profession during or before your pregnancy?

______________________________

**How much did you work?**

- ☐ Full-time
- ☐ Part-time with |__|__|,|__| %

**Did you often have to work overtime?**

- ☐ Yes
- ☐ No

If yes, how many hours a week on average?

☐ < 2 h ☐ 2–5 h ☐ > 5 h

**How do you rate your job satisfaction at that time?**

☐ Very good ☐ Good ☐ Satisfactory
☐ Sufficient ☐ Not sufficient

**Would you choose your profession again?**

☐ Yes ☐ Probably
☐ Probably not ☐ No

**Did you work in shifts?**

- ☐ Yes
- ☐ No

**Did you work night shifts?**

- ☐ Yes
- ☐ No

**Did you have the opportunity and time to take regular meals?**

☐ Never ☐ Rarely
☐ Mostly ☐ Always

**Did you suffer from insomnia at the time?**

- ☐ Yes
- ☐ No

**How many hours of sleep per night did you get on average before your pregnancy?**

☐ < 5 h ☐ 5–7 h ☐ > 7 h

**Would you describe your day-night rhythm before pregnancy as "undisturbed"?**

- ☐ Yes
- ☐ No

**Were you able to sleep through the night without your sleep being disturbed regularly (e.g. by street noise, neighbors, children etc.)?**

- ☐ Yes
- ☐ No

**How many children lived in your household before or during pregnancy?**

Number: |__|__|

**What was your marital status at the time of pregnancy?**

☐ Single ☐ Married ☐ Widowed
☐ Divorced ☐ Steady partnership

**Do you generally cope well with stress?**

- ☐ Yes
- ☐ No

**Do you suffer from any type of mental illness (depression, anxiety disorders, etc.)?**

- ☐ Yes
- ☐ No

**How healthy did you feel before the pregnancy?**

☐ Healthy ☐ Healthy, temporary slight impairments
☐ Not healthy ☐ Healthy, minor permanent impairments

**Were you active in other areas in your free time?**
(please simply tick)

| | Several times a week | Once a week | Rarely | Never |
|---|---|---|---|---|
| Running | ☐ | ☐ | ☐ | ☐ |
| Swimming | ☐ | ☐ | ☐ | ☐ |
| Cycling | ☐ | ☐ | ☐ | ☐ |
| Walking/ Hiking | ☐ | ☐ | ☐ | ☐ |
| Weights | ☐ | ☐ | ☐ | ☐ |
| Others | ☐ | ☐ | ☐ | ☐ |
