# Supplementary material for: Circadian rhythm and gestational diabetes: working conditions, sleeping habits and lifestyle influence insulin dependency during pregnancy
Source: Acta Diabetol. 2021 Apr 10;58(9):1177–86. doi: 10.1007/s00592-021-01708-8 (PMC8316165; doi:10.1007/s00592-021-01708-8)
Supplement: Supplementary file 2 — Supplementary file2 (DOCX 26 KB) [file 592_2021_1708_MOESM2_ESM.docx]

| Supplemental Table S2 - Pregnancy characteristics and perinatal outcome of the treatment groups: Diet, Bolus, MDI and Basal | | | | | | |
| --- | --- | --- | --- | --- | --- | --- |
| Variable | Total cohort  (n=235) | 'Diet‘  (n=132) | 'Bolus‘  (n=25) | 'MDI‘  (n=39) | 'Basal‘  (n=39) | p* |
| Hba1c at delivery in % | 5.5 (5.2; 5.7) | 5.5 (5.2; 5.7) | 5.5 (5.3; 5.8) | 5.5 (5.2; 5.8) | 5.4 (5.2; 5.6) | n.s. |
| Hba1c at delivery in mmol/mol | 37 (33; 39) | 37 (33; 39) | 37 (34; 40) | 37 (33; 40) | 36 (33; 38) | n.s. |
| Hba1c changes in % | 0.2 (0; 0.4) | 0.2 (0.1; 0.4) | 0.2 (0; 0.4) | 0 (-0.2; 0.1) | 0.1 (0.1; 0.4) | **<0.01** |
| HbA1c changes in mmol/mol | 2.2 (0;4.4) | 2.2 (1.1-4.4) | 2.2 (0; 4.4) | 0 (-2.2; 1.1) | 1.1 (1.1;4.4) | <0.01 |
| Insulin IU/kg | 0.31 (0.17; 0.48) |  | 0.27 (0.19; 0.37) | 0.48 (0.34; 0.61) | 0.17 (0.12; 0.29) | **<0.01** |
| GWG in kg | 12 (8; 16.6) | 12 (8; 16.4) | 12 (8.3; 17.2) | 12.9 (8; 16.6) | 14 (9; 16.4) | n.s. |
| Pregnancy complications | 31.3 % | 31.3 | 38.9 | 31.3 | 27.3 | n.s. |
| Pre-eclampsia/ PIH/ HELLP | 8.3% | 6.3 | 6.7 | 10.7 | 13.8 | n.s. |
| IOL | 39% | 40.5% | 33.3% | 27.3% | 50% | n.s. |
| C-section | 32% | 27.4% | 40.9% | 42.9% | 31.3% | n.s. |
| Birth weight | 3500 (3135; 3825) | 3493 (3047; 3725) | 3455 (3265; 3878) | 3660 (3150; 3900) | 3455 (3265; 3878) | n.s. |
| GA at delivery | 39 (38; 40) | 39 (38; 40) | 39 (38; 40) | 39 (38; 40) | 39 (39; 40) | n.s. |
| Voigt’s Percentile | 58 (31; 78) | 54 (26; 72) | 58 (35; 83) | 73 (42; 93) | 58 (35; 83) | **<0.05** |
| SGA | 6% | 7.8% | 10% | 0% | 3.3% | n.s. |
| LGA | 12.6% | 7.8% | 5% | 32.4% | 13.3% | **<0.01** |
| 5min APGAR | 9 (9;10) | 9 (9; 10) | 9 (9;10) | 9 (9; 10) | 9 (9; 10) | n.s. |
| pH | 7.26 (7.20; 7.31) | 7.26 (7.20; 7.31) | 7.27 (7.21; 7.32) | 7.26 (7.18; 7.31) | 7.27 (7.21; 7.32) | n.s. |
| NICU admission | 7% | 5.4% | 6.3% | 9.7% | 10.7% | n.s. |
| hyperbilirubinemia | 27.3% | 25% | 25% | 37.5% | 26.1% | n.s. |
| hypoglycemia | 1.7% | 0% | 0% | 7.1% | 3.6% | n.s. |

Data are n (%) or median (interquartile range) unless otherwise specified. * p - Comparison of the four subgroups using Wilcoxon test, p<0.05 is significant and bold. GA – gestational age; GWG – gestational weight gain; IOL – induction of labor; LGA – large for gestational age; NICU – neonatal intensive care unit; PIH – pregnancy induced hypertension; SGA – small for gestational age;
